# Mechanism of KLF4 Protection against Acute Liver Injury via Inhibition of Apelin Signaling

**DOI:** 10.1155/2019/6140360

**Published:** 2019-10-10

**Authors:** Weitao Ji, Hongyun Shi, Hailin Shen, Jing Kong, Jiayi Song, Hongyan Bian, Xinrui Lv

**Affiliations:** ^1^The First Affiliated Hospital of Henan University, Kaifeng, Henan, China; ^2^Department of Oncology, The Affiliated Hospital of Hebei University, Baoding, Hebei, China; ^3^Key Laboratory of Receptors-Mediated Gene Regulation and Drug Discovery, School of Basic Medicine, Henan University, Kaifeng, Henan, China

## Abstract

Krüppel-like factor 4 (KLF4) is a key transcription factor that regulates genes involved in the proliferation or differentiation in different tissues. Apelin plays roles in cardiovascular functions, metabolic disease, and homeostatic disorder. However, the biological function of apelin in liver disease is still ongoing. In this study, we investigated the mechanism of KLF4-mediated protection against acute liver injury via the inhibition of the apelin signaling pathway. Mice were intraperitoneally injected with carbon tetrachloride (CCl_4_; 0.2 mL dissolved in 100 mL olive oil, 10 mL/kg) to establish an acute liver injury model. A KLF4 expression plasmid was injected through the tail vein 48 h before CCl_4_ treatment. In cultured LX-2 cells, pAd-KLF4 or siRNA KLF4 was overexpressed or knockdown, and the mRNA and protein levels of apelin were determined. The results showed that the apelin serum level in the CCl_4_-injected group was higher than that of control group, and the expression of apelin in the liver tissues was elevated while KLF4 expression was decreased in the CCl_4_-injected group compared to the KLF4-plasmid-injected group. HE staining revealed serious hepatocellular steatosis in the CCl_4_-injected mice, and KLF4 alleviated this steatosis in the mice injected with KLF4 plasmid. *In vitro* experiments showed that tumor necrosis factor-alpha (TNF-*α*) could downregulate the transcription and translation levels of apelin in LX-2 cells and also upregulate KLF4 mRNA and protein expression. RT-PCR and Western blotting showed that the overexpression of KLF4 markedly decreased basal apelin expression, but knockdown of KLF4 restored apelin expression in TNF-*α*-treated LX-2 cells. These *in vivo* and *in vitro* experiments suggest that KLF4 plays a key role in inhibiting hepatocellular steatosis in acute liver injury, and that its mechanism might be the inhibition of the apelin signaling pathway.

## 1. Introduction

The liver is a crucial organ with metabolic and detoxification functions. Acute liver injury can arise from multiple factors, including viral infection, trauma, or chemical reagents, such as alcohol, drugs, and toxic substance [1, 2]. Serious or continuous liver injury leads to liver fibrosis, cirrhosis, and even the development of hepatocellular carcinoma cells (HCC) [3, 4]. Although the pathogenic factors and mechanisms of acute liver injury have been widely reported, the true nature of liver injury is still far from being well understood.

Krüppel-like factor 4 (KLF4) is a multifunctional, zinc-finger transcription factor that regulates genes involved in the cell cycle, proliferation, differentiation, apoptosis, and responses to external stress. Intriguingly, it is not only a tumor suppressor but also an oncogene in different tumor tissues where it regulates the expression of various genes [5, 6]. Studies have confirmed that KLF4 can regulate pathological processes such as liver fibrosis and HCC formation [7], and a recent study reported that KLF4 promoted HepG2 cell scattering induced by hepatocyte growth factor [8]. The expression of KLF4 at both the protein and mRNA levels is drastically reduced in HCC tissues and all human HCC cell lines when compared with normal human liver tissues and hepatocyte lines [9, 10]. Additionally, KLF4 can reduce migration and invasion by HCC cells via the upregulation of tissue inhibitor of metalloproteinase (TIMP)-1 and TIMP-2 [11]. However, the function of KLF4 in acute liver injury remains unclear.

It has been demonstrated that apelin plays important and varied roles in the physiology and pathophysiology of many organs, including the regulation of blood pressure, cardiac contraction, angiogenesis, metabolic balance, cell proliferation, apoptosis, and inflammation [12–16]. One study reported that the expression of apelin increases sharply in the liver tissues of cirrhotic human and rats when compared with that of control groups [17]. In addition, circulating levels of apelin markedly increase in human and rats with cirrhosis [18]. The expression of apelin is also enhanced under hypoxic or proinflammatory conditions in human hepatic stellate cells (HSCs), and it promotes liver fibrosis or cirrhosis progression [19, 20]. Apelin expression is upregulated in a murine HCC tumor model and in clinical specimens [21, 22]. However, the mechanism of the upregulation of apelin expression in liver disease was still under investigation. In the present study, we aimed to investigate the function and mechanism of KLF4 in protection against liver injury via the inhibition of apelin signaling.

## 2. Materials and Methods

### 2.1. Animal Experiments

C57BL/6 mice (SPF, male, 6 to 8 weeks old, 22–24 g) were purchased from the Institutional Animal Care and Use Committee of Charles River (Beijing, China, License number: SCXK 2012-0001). The mice were bred and housed in a specific pathogen-free environment at the Key Laboratory of Receptors-Mediated Gene Regulation and Drug Discovery of School of Basic Medicine, Henan University, and all procedures performed in studies involving animals were carried out in accordance with The Code of Ethics of the World Medical Association (Declaration of Helsinki) and the ethical standards of Animal Research Ethics Committee of Henan University. The mice were intraperitoneally injected with a mixture of carbon tetrachloride (0.2 mL CCl_4_ dissolved in 100 mL of olive oil) at a dose of 10 mL/kg body weight. The mice of the normal control group received an intraperitoneal injection of the same volume of olive oil as the CCl_4_ group. The mice were sacrificed at 24 h after the CCl_4_ injection. The KLF4 plasmid (10 mg/kg) was biosynthesized by Invitrogen and was injected into tail veins 48 h before CCl_4_ exposure, and the GFP plasmid vector was injected into the tail veins of the animals in the control group at the same time.

### 2.2. Cell Culture and Treatment

LX-2 cells were purchased from Meixuan Biological Science and Technology Ltd. (Shanghai, China). Cells were seeded in 60 mm plates and grown to confluence for 24 h in high-glucose Dulbecco's modified Eagle's medium (DMEM) supplemented with 10% fetal calf serum (FBS) in a humidified atmosphere containing 5% CO_2_ at 37 °C. Thereafter, cells were switched to serum-free DMEM for 24 h and then treated with TNF-*α* (10 ng/mL; Sigma-Aldrich) for the indicated times.

### 2.3. Hematoxylin-Eosin (HE) Staining

Liver tissues of mice were fixed in 10% neutral buffered formalin at room temperature (RT) for 24 h and then embedded in paraffin and sectioned to a thickness of approximately 5 *μ*m. Sections were stained with HE following standard procedures and examined by light microscopy.

### 2.4. Serum Alanine Aminotransferase (ALT) and Aspartate Aminotransferase (AST) Level Measurement

Blood from mice was collected in a common tube from the angular vein when all the mice were sacrificed. Samples were left to stand for 4–6 h at RT and then centrifuged at 3000 *g* for 5 min. The serum was collected, and ALT and AST levels were determined by the Reitman Frankel method using commercially available assay kits (Jiancheng Bioengineering Institute, Nanjing, China).

### 2.5. Immunohistochemistry

Mouse liver tissues were fixed with 10% neutral buffered formalin at RT for 24 h and then embedded in paraffin and sectioned to a thickness of approximately 5 *μ*m. Sections were immunostained using a standard protocol. The immunostaining of sections was performed with anti-apelin, anti-KLF4, and anti-cyclin D1 antibodies (1 : 100 dilution), and sections were also counterstained with haematoxylin. Staining intensities were determined by measurement of the integrated optical density following examination by light microscopy and by using Image-Pro Morphometric System software in a double-blind manner.

### 2.6. RNA Preparation and Quantitative Reverse Transcription-PCR (qRT-PCR)

Total RNA was isolated using TRIzol® reagent (Invitrogen) according to the manufacturer's instructions. RNA concentrations and purity were determined by measuring the 260/280 nm absorbance ratio. GAPDH (glyceraldehyde-3-phosphate dehydrogenase) gene primers were used as an internal control for RNA template normalization. Quantitative PCR was performed using a Platinum SYBR Green qPCR Super Mix UDG Kit (Invitrogen and the following primers were: apelin (mouse) 5′ TCTTGGCTCTTCCCTCTTTTCA 3′ (sense) and 5′ GTGCTGGAATCCACTGGAGAA 3′ (antisense), KLF4 (mouse) 5′  CAGCTGGCAAGCGCTACA 3′ (sense) and 5′ CCTTTCTCCTGATTATCCATTC 3′ (antisense), cyclin D1 (mouse) 5′ GCGTACCCTGACACCAATCTC 3′ (sense) and 5′ CTCCTCTTCGCACTTCTGCTC 3′ (antisense), TNF-*α* (mouse) 5′ AACGGGGAAGCAACTTAGCA 3′ and 5′ ACCACAGGGCAAAGGAGATT 3′, GAPDH (mouse) 5′ TGTGAACGGATTTGGCCGTA 3′ (sense) and 5′ ACTGTGCCGTTGAATTTGCC 3′ (antisense), apelin (human) 5′ GCTCTGGCTCTCCTTGACC 3′ (sense) and 5′ CCATTCCTTGACCCTCTGG 3′ (antisense), KLF4 (human) 5′ CGGACATCAACGACGTGAG 3′ (sense) and 5′ GACGCCTTCAGCACGAACT 3′ (antisense), and GAPDH (human) 5′ GGAGCGAGATCCCTCCAAAAT 3′ (sense) and 5′ GGCTGTTGTCATACTTCTCATGG 3′ (antisense). The relative expression level was calculated using the following equation: relative gene expression = 2^−*∆∆*CT^.

### 2.7. Western Blotting

Crude proteins were extracted from liver tissues of mice or LX-2 cells as described previously [23], resolved by SDS/PAGE, and then transferred to a PVDF membrane (Millipore). Membranes were blocked with 5% (*w*/*v*) nonfat dried skimmed milk powder in TTBS buffer (100 mM Tris/HCl, pH 7.5, 150 mM NaCl, and 0.5% Tween 20) for 2 h at 37°C and then incubated overnight at 4°C with the following primary antibodies: 1 : 300 dilution rabbit anti-apelin (GeneTex), 1 : 500 dilution rabbit anti-KLF4 (Abcam), 1 : 2500 dilution rabbit anti-cyclin D1 (Abcam), and anti-*β*-actin and rabbit anti-IgG (Santa Cruz Biotechnology). After incubation with the appropriate secondary antibody, the immunoreactive signals of antibody-antigens were visualized using a Chemiluminescence Plus Western Blot Analysis kit (Santa Cruz Biotechnology).

### 2.8. Immunofluorescence Staining

LX-2 cells were fixed in 4% paraformaldehyde and permeabilized with 0.1% Triton X-100 and then incubated with anti-apelin or anti-KLF4 antibody and further stained with FITC-conjugated secondary antibody. Staining with 4′,6-diamidino-2-phenylindole (DAPI) was used to visualize nuclear localization. Each section was examined under an inverted fluorescence microscope (Leica).

### 2.9. Adenovirus Infection

PAd-KLF4 was kindly provided by Dr. Wen Jin-Kun (The Key Laboratory of Neural and Vascular Biology, China Administration of Education, Hebei Medical University). The adenovirus was amplified by infecting A293 cells, and culture supernatant with a titer of 3 × 10^8^ pfu/mL was used to infect LX-2 cells. After adenovirus delivery, KLF4 were detected using RT-PCR and Western blot analysis.

### 2.10. SiRNA Transfection

Small-interfering RNA (siRNA) targeting human KLF4 (si-KLF4) and a nonspecific siRNA (si-NS) were purchased from Santa Cruz Biotechnology. Transfection was performed using Lipofectamine™ reagent (Invitrogen) following the manufacturer's instructions. At 24 h following transfection, LX-2 cells were incubated with or without TNF-*α* (10 ng/mL). Cells were then harvested and used for RT-PCR and Western blotting assays.

### 2.11. Statistical Analyses

Data are presented as means ± S.E.M. from three or more independent experiments. Statistical analyses were performed using the Student's *t*-test or one-way ANOVA depending on the number of groups compared. Differences were considered significant at *P* < 0.05.

## 3. Results

### 3.1. Differential Expression of Apelin and KLF4 in CCl_4_-Induced Acute Liver Injury in Mice

**Figure 1:**
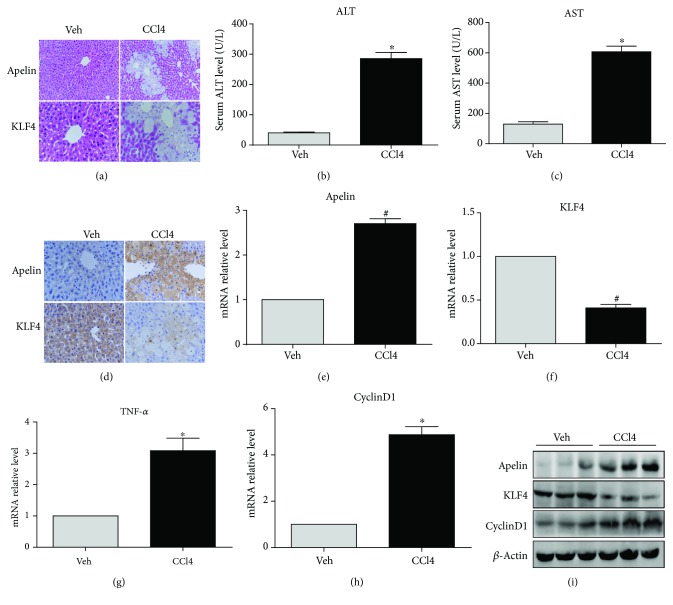
Differential expression of apelin and KLF4 in CCl_4_-induced acute liver injury. Mice received an intraperitoneal injection of carbon tetrachloride (0.2 mL CCl_4_ dissolved in 100 mL olive oil) at a dose of 10 mL/kg body weight. The mice in the control group were injected with oil only. (a) Representative photomicrographs of liver histology (HE staining; magnification: upper ×200; lower ×400). (b, c) Serum ALT and AST levels. ^∗^*P* < 0.01 compared with the vehicle (Veh) group. (d) Immunohistochemical staining of apelin and KLF4 protein following to olive oil and CCl_4_ treatment (magnification: upper ×200; lower ×400); (e–h) mRNA expression of apelin, KLF4, TNF-*α*, and cyclinD1 in CCl_4_-induced mice. ^#^*P* < 0.05 or ^∗^*P* < 0.01 compared with the Veh group. (i) Protein expression of apelin, KLF4, and cyclin D1 in CCl_4_-induced mice. *β*-Actin was used as a loading control.

The murine acute liver injury model was established by single intraperitoneal injections of CCl_4_. HE staining showed that lobular structure of liver tissue from mice treated only with olive oil treatment was clear and that the hepatic cells were arranged in neat rows (Figure 1(a)). In contrast, hepatocellular steatosis was present around the central veins of hepatic lobules in CCl_4_-treated mice. Alanine aminotransferase (ALT) and aspartate transaminase (AST) levels in serum were significantly elevated after intraperitoneal injection of CCl_4_ (Figures 1(b) and 1(c)). Immunohistochemical staining indicated that the expression of apelin protein increased, while KLF4 protein expression decreased, in the hepatocytes of mice treated with CCl_4_ (Figure 1(d)). Protein levels of apelin and cyclin D1 (a cell cycle marker protein) and TNF-*α* mRNA and protein levels were upregulated, while KLF4 mRNA and protein levels were downregulated after CCl_4_ injection (Figures 1(e)–1(i)). These results indicate that apelin and KLF4 involved the hepatic stestosis in CCl_4_-induced acute liver injury.

### 3.2. KLF4 Protects Mice against CCl_4_-Induced Acute Liver Injury by Inhibiting Apelin Signaling

**Figure 2:**
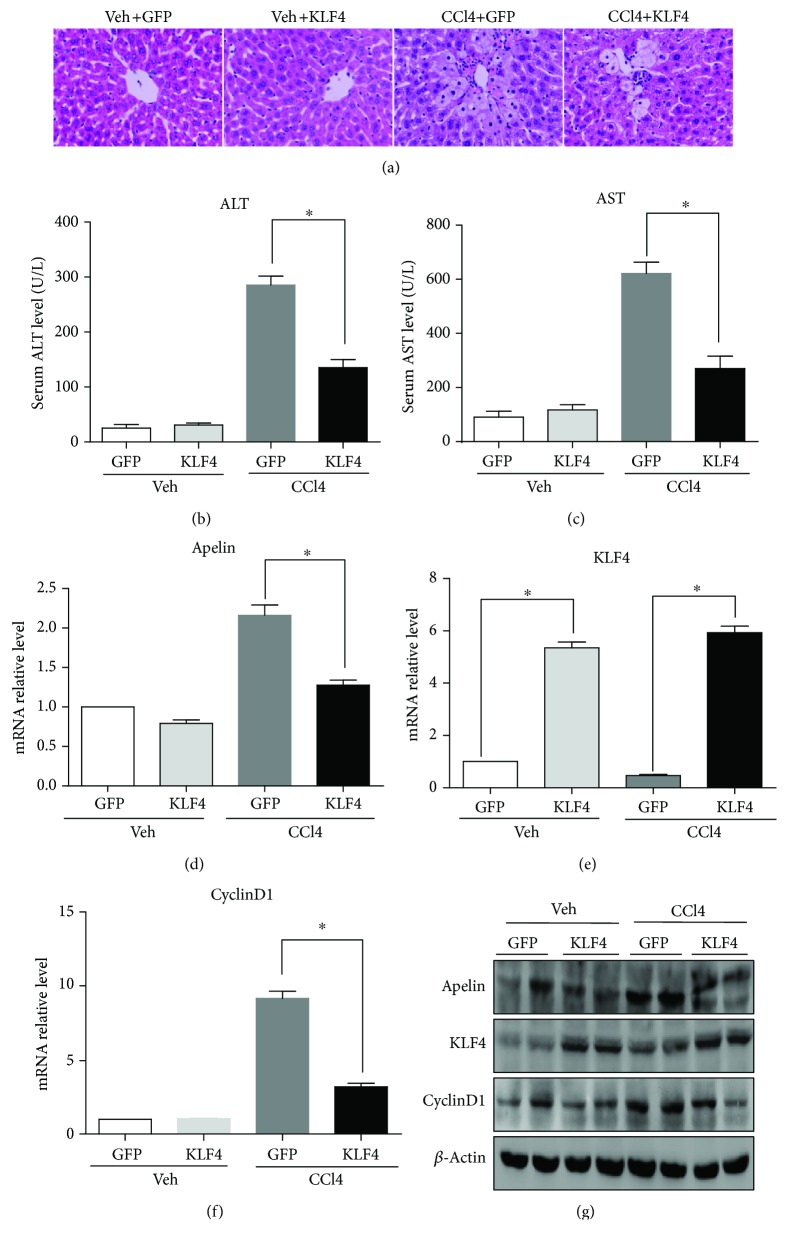
KLF4 protects mice against CCl_4_-induced acute liver injury. KLF4 (10 mg/kg) or GFP (10 mg/kg, control) plasmids were injected into the tail vein of mice 48 h before CCl_4_ injection. Control mice were injected with olive oil vehicle (Veh) lacking CCl_4_. The mice were sacrificed 24 h after CCl_4_ treatment. (a) HE stained sections were produced from liver tissues of the Veh and KLF4 groups (magnification: ×400). (b, c) Serum ALT and AST levels were determined in the different groups.^∗^*P* < 0.01 compared with the GFP group. (d–f) The mRNA expression of apelin, KLF4, and cyclinD1 was determined by qRT-PCR analysis of liver tissue from the different groups of mice. ^∗^*P* < 0.01 compared with the GFP group. (g) Crude proteins were extracted from the liver tissues and analyzed by Western blotting using anti-apelin, anti-cyclin D1, and anti-KLF4 antibodies. *β*-Actin was used as a loading control.

**Figure 3:**
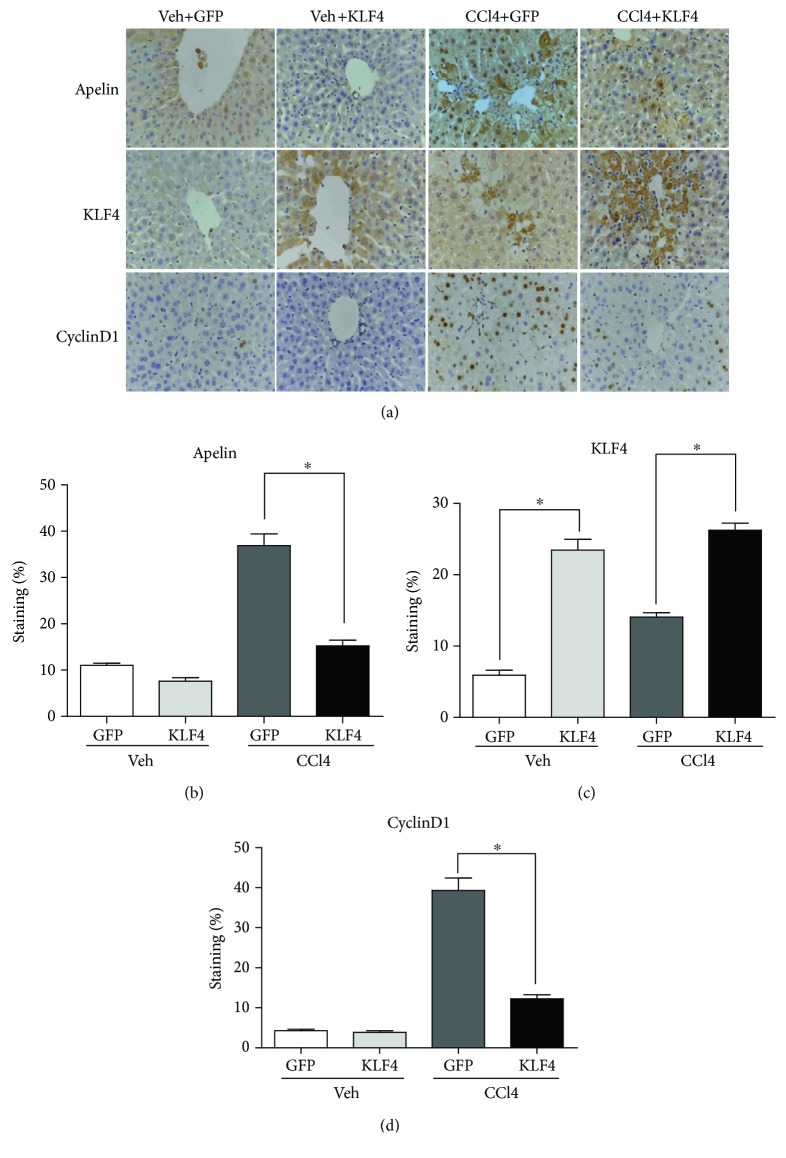
The effects of KLF4 on immunohistochemical staining of hepatic tissue from CCl_4_-induced, acutely injured livers. (a) Immunohistochemical staining of apelin, KLF4, and cyclinD1 protein in different groups of mice (magnification: ×400). The apelin, KLF4, and cyclinD1 indices were calculated as follows: positive cells/total intimal cells. (b–d) Quantified results are given as means ± S.E.M of four independent experiments. ^∗^*P* < 0.01 compared with GFP group. (*n* = 6 in each group).

To evaluate whether KLF4 plays a protective role in CCl_4_-induced acute liver injury in mice, the KLF4 plasmid was injected into the tail vein 48 h prior to CCl_4_ treatment and the mice were sacrificed after 24 h of CCl_4_ treatment. The mice that received the KLF4 plasmid treatment exhibited less hepatic necrosis than those that had only been exposed CCl_4_ and the control GFP plasmid (Figure 2(a)). In addition, serum ALT and AST levels were significantly lower in the KLF4 plasmid-treated animals (Figures 2(b) and 2(c)). These results demonstrate that KLF4 can decrease hepatic necrosis and protect mice against CCl_4_-induced acute liver injury. Furthermore, RT-PCR and Western blotting analysis also revealed that mRNA and protein levels were substantially higher in the liver tissues of KLF4-overexpressing mice than in the null plasmid mice after CCl_4_ treatment (Figures 2(e) and 2(g)). These results confirmed that a KLF4 overexpression liver model had been successfully established in mice. Simultaneously, the results demonstrated lower expression of apelin in the KLF4-overexpressing mice than in the null plasmid mice after CCl_4_ treatment (Figures 2(d) and 2(g)). These results suggest that KLF4 can decrease apelin expression and so protect mice against CCl_4_-induced acute liver injury. Additionally, paraplastic hepatocytes are one of the main causes of liver fibrosis, and RT-PCR and Western blotting analyses showed that cyclin D1 expression levels were apparently lower in the KLF4-overexpressing mice than in those treated with the null plasmid prior to CCl_4_ administration (Figures 2(f) and 2(g)). Finally, immunohistochemical staining showed that the expression of apelin, KLF4, and cyclin D1 in different groups was concordant with the RT-PCR and Western blotting results (Figures 3(a)–3(d)).

### 3.3. TNF-*α* Decreases Apelin mRNA and Protein Levels, but Increases KLF4 Expression in LX-2 Cells

**Figure 4:**
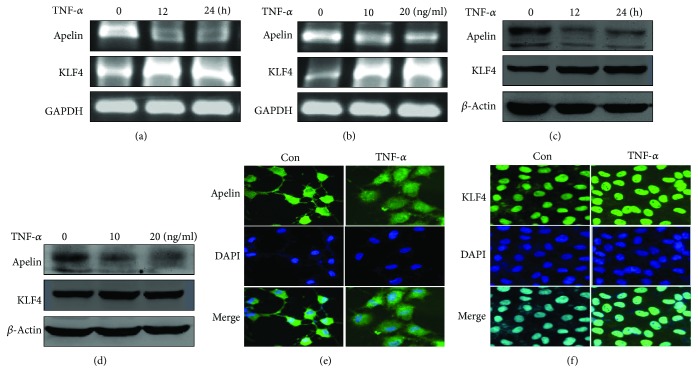
TNF-*α* decreases apelin mRNA and protein levels, but increases KLF4 expression in LX-2 cells. All experiments were performed in triplicate. (a, b) LX-2 cells were treated with TNF-*α* (10 ng/mL) for various times or at different doses (for 24 h). Total RNA was transcribed with reverse transcriptase and amplified by PCR. GAPDH was used as an internal control. (c, d) Western blotting was performed using anti-apelin and anti-KLF4 antibodies to examine protein expression at various times or at different doses. *β*-Actin was used as a loading control. (e) LX-2 cells were treated with TNF-*α* (10 ng/mL) for 24 h and the cells were then fixed and stained by immunofluorescence. Expression of apelin was detected in the cytoplasm (magnification: ×200). (f) LX-2 cells were treated with TNF-*α* (10 ng/mL) for 24 h and then the cells were then fixed and stained by immunofluorescence. The expression of KLF4 was detected in nuclei (magnification: ×200).

LX-2 cells, which are a type of HSC, and activated HSC play an important role in both acute liver injury and fibrosis. RT-PCR and Western blotting methods were used to evaluate the expression levels of apelin and KLF4 in LX-2 cells after TNF-*α*-stimulation. Both apelin mRNA and protein levels decreased in a time-dependent manner when LX-2 cells were treated with 10 ng/mL TNF-*α*. Moreover, TNF-*α* also dose-dependently decreased apelin mRNA and protein levels (Figures 4(a)–4(d)). Immunofluorescence staining showed that apelin was localized in the cytoplasm of LX-2 cells and that stimulation by TNF-*α* for 24 h decreased apelin basal expression (Figure 4(e)). In contrast, TNF-*α* treatment of LX-2 cells increased both KLF4 mRNA and protein levels in a time- and dose-dependent manner (Figures 4(a)–4(d)). Immunofluorescence staining showed an increase in the levels of KLF4, which was mainly localized to the nucleus (Figure 4(f)). These results demonstrated that TNF-*α* downregulated apelin gene expression, but upregulated KLF4 expression *in vitro*.

### 3.4. KLF4 Protects Hepatocytes by Inhibiting Apelin Signaling *In Vitro*

**Figure 5:**
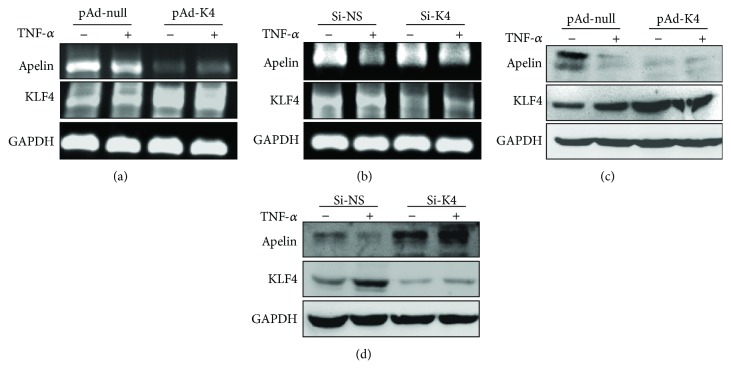
KLF4 plays a negative regulatory role on apelin expression in LX-2 cells. All experiments were performed in triplicate. (a, b) LX-2 cells were infected with pAd-null or pAd-KLF4 for 24 h or were transfected with si-NS or si-KLF4 for 24 h. Cells were then treated with TNF-*α* (10 ng/mL) for 24 h. Total cellular RNA was transcribed with reverse transcriptase and amplified by PCR. GAPDH was used as an internal control. (c, d) Crude proteins extracted from the treated cells were analyzed using Western blotting with anti-apelin, anti-KLF4, and anti-*β*-actin antibodies. *β*-Actin was used as a loading control.

To further verify the importance of KLF4 in regulating apelin expression, LX-2 cells were infected with pAd-KLF4 or transfected with si-KLF4 to overexpress or knock down endogenous KLF4 expression, respectively. RT-PCR and Western blotting showed that the overexpression of KLF4 markedly decreased basal apelin expression at both transcription and translation levels (Figures 5(a)–5(d)). Conversely, knockdown of KLF4 recovered the decrease in apelin expression induced by TNF-*α*, whereas si-NS had no impact on apelin expression. These results suggest that KLF4 can negatively regulate apelin expression in LX-2 cells via TNF-*α*.

## 4. Discussion

Previous studies had confirmed that the pathogenesis of acute liver injury involved a series of different of cell signaling pathways [24–27]. However, the nature of acute liver injury still remains mainly unelucidated. The present study clearly demonstrates, for the first time, that KLF4 can protect against liver injury by inhibiting apelin signaling in CCl_4_-induced acute liver injury model. Apelin is highly expressed in the lung, heart, mammary gland, brain, kidney, testicular, and ovarian tissues [28, 29], but exhibits lower expression levels in normal liver [30]. Principe et al. reported that apelin expression increases sharply in the hepatic tissue of cirrhotic rats compared to that of controls [17]. Furthermore, the circulating levels of apelin are markedly increased in rats with cirrhosis. Other studies have reported that apelin can promote Fas-induced liver injury via activation of JNK cell signaling, and it has been demonstrated that liver apoptosis and injury is significantly alleviated in APJ^−/−^ mice compared with wild-type animals [31]. Our results demonstrate that apelin expression exhibits a sharp rise upon CCl_4_-induced acute liver injury in mice (Figures 1(d), 1(e), and 1(i)). Additionally, some studies have reported that serum levels of apelin were increased in some liver diseases, such as nonalcoholic fatty liver disease and cirrhosis [32, 33]. The results presented here further indicate that apelin participates in hepatic stestosis in CCl_4_-induced acute liver injury.

These results also demonstrate that KLF4 mRNA and protein levels are downregulated following CCl_4_-induced acute liver injury in mice. KLF4, a gut-enriched GKLF and zinc-finger transcription factor, regulates a multitude of processes in cell growth and development, proliferation and differentiation, inflammation, and apoptosis, and is even both a tumor suppressor and oncogene. A recent study reported that the expression of KLF4 declines dramatically in activated rat HSCs in the liver tissues of cirrhotic patients [34]. To determine whether KLF4 protects against CCl_4_-induced acute liver injury, a plasmid containing KLF4 was injected via tail veins 48 h prior to CCl_4_ treatment. Histological examination showed that the mice pretreated with the KLF4 plasmid exhibited less hepatic necrosis in their liver tissue (Figure 2(a)) and significantly decreased serum ALT and AST levels (Figures 2(b) and 2(c)). Furthermore, KLF4 mRNA and protein levels were substantially higher in the liver tissues of KLF4 plasmid-treated mice than those of their null plasmid-treated counterparts. These results confirm that a murine hepatic KLF4 overexpression model had been successfully established. Previous studies have shown that the expression of KLF4 is lower in HCC tissues or cell lines than in normal liver tissues or cells [35, 36], and that KLF4 can inhibit HCC cell proliferation and metastasis [37]. Hyperplasia of hepatocytes is one of the main causes of liver fibrosis, which is consistent with our observations that the expression level of cyclin D1 is apparently lower in CCl_4_-treated KLF4-overexpressing mice than in those pretreated with the null plasmid. Furthermore, a study showed that the knockdown of KLF4 can significantly enhance apoptosis in the liver cell line BNCL.2 [38], indicating that KLF4 can protect against liver injury by suppressing hepatocyte growth, metastasis, and apoptosis.

Our results also show that, following CCl_4_ treatment, the expression and serum levels of apelin in the KLF4-overexpressing mice are lower than in those pretreated with the null plasmid. These results in part demonstrate that KLF4 can decrease hepatic necrosis and protect against liver tissue injury by decreasing apelin expression. A recent study showed that KLF4 can inhibit HCC cell growth and metastasis by the downregulation of Micro-135a-5p by TGF-*β*1 [39]. It has been reported that KLF4 negatively regulates the epithelial-mesenchymal transition (EMT) of gastrointestinal cancers through crosstalk between the TGF-*β*, Notch, and Wnt signaling pathways [40]. Additionally, other *in vitro* and *in vivo* experiments have confirmed that KLF4 can revert the EMT by suppressing slug expression and that overexpression of KLF4 can reduce HCC cell migration and invasion [41].

Although some studies have indicated that TNF-*α* participates in the ontogeny and development of liver injury [42], TNF-*α* can also repair liver injury by suppressing cell death, activating stem cells, and promoting epithelial proliferation in the pathological process of liver injury [43–45]. The results of this study also suggest that TNF-*α* might play a dual role in CCl_4_-induced liver injury [46]. Previous studies have shown that apelin is overexpressed in HSCs from cirrhotic rats, and that the serum level of apelin is higher in patients or rats with cirrhosis than that in normal individuals [17, 20, 47]. It has been shown that apelin not only powerfully induces proliferation in LX-2 cells but also promotes the synthesis of angiotension-1 (Ang-1), which is a potent proangiogenic factor and contributes to the development of liver injury and fibrosis [47]. The same study showed that the expression of apelin is downregulated in LX-2 cells upon stimulation by TNF-*α*, although the mechanism underlying this effect still remains unknown. Our *in vitro* experimental results show that TNF-*α* not only downregulates apelin expression but also upregulates KLF4 expression at both the mRNA and protein levels in LX-2 cells (Figures 4(a)–4(f)). To further verify the importance of KLF4 in regulating apelin expression, LX-2 cells were either infected with pAd-KLF4 or transfected with si-KLF4 to overexpress or knock down endogenous KLF4, respectively. RT-PCR and Western blotting showed that the overexpression of KLF4 markedly decreased basal apelin expression, while knockdown of KLF4 reversed the decrease in apelin expression induced by TNF-*α*. These results suggest that KLF4 can negatively regulate apelin expression induced by TNF-*α* in LX-2 cells. A recent study showed that the silencing of KLF4 in LX-2 cells significantly decreases the expression levels of TNF-*α*, while significantly upregulating MMP-2 expression [48]. Furthermore, the expression of KLF4 increases substantially upon TNF-*α*-induced LX-2 cells apoptosis [49].

In conclusion, we speculate that hepatic apelin acts as a proinflammatory and neoangiogenic factor and so plays an important role in the initiation and maintenance of the inflammatory and hyperplastic processes that occur in acute liver injury. KLF4 exerts a protective effect against injury liver by inhibiting apelin signaling. This study therefore identifies a possible new target pathway for the prevention and therapy of acute liver injury.

## Data Availability

The raw data used to support the findings of this study are available from the corresponding author upon request.
